# Incidence of Notifiable Diseases Among American Indians/Alaska Natives — United States, 2007–2011

**Published:** 2015-01-16

**Authors:** Nelson Adekoya, Benedict Truman, Michael Landen

**Affiliations:** 1 Office of the Director, National Center for HIV/AIDS, Viral Hepatitis, STD, and TB Prevention, CDC; 2 New Mexico Department of Health, Santa Fe, New Mexico

American Indian/Alaska Native (AI/AN) populations experience substantial disparities in the incidence of multiple diseases compared with other racial/ethnic groups in the United States. A major goal of *Healthy People 2020* is to eliminate health disparities, monitor disease trends, and identify population groups and diseases for targeted interventions (1). High rates of certain infectious diseases continue to be a major problem facing AI/AN populations (2). During 1990–2011, incidence rates for some infectious diseases declined among AI/AN populations, but disparities remain and AI/AN populations are still disproportionately affected (2,3). To describe disparities in selected notifiable diseases among AI/ANs, CDC analyzed data from the National Notifiable Diseases Surveillance System (NNDSS) for 2007–2011, the most recent 5 years for which data are available. The results of this analysis of 26 infectious diseases indicate that incidence rates of 14 diseases were higher for AI/ANs than for whites. Interventions are needed to address and reduce disparities in chlamydia, gonorrhea, West Nile virus, spotted fever rickettsiosis, and other infections among AI/ANs.

NNDSS is a public health surveillance system that collects data on nationally notifiable diseases in the United States and its territories (3). CDC maintains the system, in collaboration with the Council of State and Territorial Epidemiologists, which determines nationally notifiable diseases and approves use of national surveillance case definitions. Each year, state epidemiologists, other Council of State and Territorial Epidemiologists members, and CDC staff members collaborate in determining for which diseases universal national case reporting yields important data and therefore should remain on the list of nationally notifiable diseases. For this study, CDC examined diseases that were notifiable during the study period and for which a minimum of 20 cases was recorded for AI/ANs. Human immunodeficiency virus/acquired immunodeficiency syndrome was excluded from the analysis because of changes in the case definition and surveillance beginning in 2009 such that data from earlier years cannot be combined with later data. Data were abstracted from a series of published annual *Summary of Notifiable Diseases* reports for sexually transmitted diseases, arboviral diseases, and tuberculosis because these diseases are not directly reported to the surveillance system. Neuroinvasive and non-neuroinvasive West Nile virus disease reports were combined in a single category as West Nile disease reports. Incidence rates for Asians/Pacific Islanders are not presented in this report because, with the exception of tuberculosis and acute viral hepatitis B, for which the rates are higher among Asians/Pacific Islanders, lower incidence rates were recorded for multiple diseases (e.g., shigellosis, salmonellosis, mumps, Lyme disease, and syphilis) compared with whites (3). However, Asians/Pacific Islanders and persons of “other race” category were included in the total counts for each disease. A person’s race in the surveillance system is based on self-reporting, regardless of ethnicity. Incidence rates were not calculated by ethnicity (i.e., Hispanic or non-Hispanic) for this report.

For 26 notifiable diseases examined for 2007–2011, a total of 12,420,236 cases were recorded (Table). Among 20 diseases for which >10,000 cases were reported nationally, incidence was higher in nine (45%) diseases for AI/ANs. Missing data on race ranged from 0.3% for *Chlamydia trachomatis* infection and 0.8% for tuberculosis to 42% for giardiasis. Race information was complete for >70% of cases for 22 of the 26 diseases. The four with incomplete data for ≥30% of cases during this period were botulism, foodborne; ehrlichiosis, total; giardiasis; and Lyme disease. Among the 22 diseases for which >70% of records had complete race information, rates were higher in 12 (55%) among AI/ANs.

Of the 12 diseases with race information for >70% of records and for which rates were higher among AI/ANs than among whites, the largest difference was for hantavirus pulmonary syndrome, which was reported 15 times more often among AI/ANs than among whites; however, only 20 cases were reported among AI/ANs of a total of 112 cases reported during 2007–2011. The second largest difference was for tularemia, which was reported 6.8 times as often among AI/ANs. There were 47 cases among AI/ANs out of a total of 626. Among more commonly reported diseases, incidence rates were 4.2 times higher among AI/ANs than whites for spotted fever rickettsiosis, 2.5 times higher for *Chlamydia trachomatis* infections, 2.4 times higher for gonorrhea, 2.1 times higher for West Nile virus, 1.9 times higher for tuberculosis, 1.8 times higher for shigellosis, 1.6 times higher for acute hepatitis C, 1.3 times higher for invasive pneumococcal infection in children aged <5 years, 1.3 times higher for *Haemophilus influenzae* type b infection, and 1.2 times higher for invasive pneumococcal infection at all ages (Table).

Of the 10 diseases with race information for >70% of records for which rates were lower among AI/ANs than among whites, the largest difference was for chickenpox (varicella) which was reported 2.6 times as often among whites as among AI/ANs. The second largest difference was for cryptosporidiosis, which was reported 2.2 times as often among whites. Lyme disease was reported 3.0 times more often among whites, but 39% of records did not include race.

## Discussion

The findings in this report document disparities in the reported incidence of selected notifiable infectious diseases among AI/ANs compared with whites. When compared with whites, AI/AN incidence rates were higher for 14 of 26 diseases. Race information was complete for >70% of cases for 22 of 26 (85%) diseases in this study, compared with a previous analysis (4) in which complete information on race was recorded for >70% of cases for only 23 of 42 diseases (55%). NNDSS has improved in coding the race variable, allowing better characterization of disease burden. It is not possible to determine if this improvement in coding is a result of several reporting jurisdictions now using the National Electronic Diseases Surveillance System (NEDSS) or a NEDSS-based compatible systems (3). With implementation of NEDSS or NEDSS-based systems, providers can rapidly transfer clinical and laboratory-based data electronically to health departments, thereby reducing the number and proportions of missing data elements. However, additional improvements might be possible when jurisdictions ascertain the value of efforts to increase the completeness of race information because laws and regulations that require public health reporting are under the purview of each reporting authority (3). The relatively low proportion of missing race data for syphilis infections and tuberculosis disease likely reflects federal and local support for more complete follow up of these cases to ensure that treatment is given and contacts are identified and treated for infection.

**What is already known on this topic?**

American Indian and Alaska Native (AI/AN) populations experience health disparities in infectious diseases. Although rates of infectious diseases have decreased among AI/AN populations, health disparities continue to exist for notifiable infectious diseases.

**What is added by this report?**

Analysis of National Notifiable Diseases Surveillance System data from 2007–2011 indicates that among 26 diseases examined, race data were included for >70% of records for 22 diseases. Among the 26, incidence rates were higher among AI/ANs than whites for 14 diseases, including chlamydia, gonorrhea, West Nile virus infection, and spotted fever rickettsiosis.

**What are the implications for public health practice?**

Jurisdictions with concentrated populations of AI/ANs might choose to routinely analyze their surveillance data and address concerns specific to this population. State and local health departments with large segments of AI/ANs have opportunities to develop efficient intervention efforts and programs tailored to this population.

In November 1990, the U.S. designated November as National American Indian Heritage Month. This proclamation calls for agencies and organizations to promote programs and activities that serve AI/ANs. Agencies and organizations have used NNDSS data to pursue *Healthy People 2020* objectives (1), to understand infectious disease trends, to assess prevention efforts, and to identify subpopulations at higher risk for multiple infections (3). When a surveillance system is used to monitor health status, program planners can identify areas in need of additional resources and efforts. To improve the accuracy of self-reported AI/AN status in NNDSS, jurisdictions with high proportions of AI/ANs might conduct sensitivity analyses, comparing the numbers of case reported to their surveillance systems with the numbers found in statewide hospital discharge systems (5,6), Indian Health Service contract health provider systems, and Indian Health Service hospital data (5). These types of analyses can validate the higher incidence rates recorded for hantavirus pulmonary syndrome and tularemia within this population and allow epidemiologists to identify specific risk factors.

Among potentially vaccine-preventable diseases, incidence rates were lower among AI/ANs than among whites for varicella, acute hepatitis A, acute hepatitis B, meningococcal disease, and pertussis; rates were slightly higher for *Haemophilus influenza* type b and invasive pneumococcal disease. These results suggest that, overall, the AI/AN population is receiving the full benefit of immunization programs.

The findings in this report are subject to at least four limitations. First, incidence rates are presented following the *Summary of Notifiable Diseases* format, which does not adjust the rates to control for differences in age, geography, and risk factors associated with the particular diseases (3). In addition, the format does not test the differences in rates for statistical significance nor explain the importance of the differences (3). Second, NNDSS is a passive surveillance system, and no attempt was made to confirm race categorization. Races might be misclassified on some records, and previous research has indicated that AI/ANs are disproportionately misclassified (5). Third, certain diseases might be underreported in NNDSS because of low priority by the state, lack of health care access of subpopulations, lack of resources for enhanced surveillance, unavailability of diagnostic tests, or many other reasons. Suboptimal reporting and completeness have been reported for several diseases of public health importance (7), but it is not known whether underreporting is equally distributed among racial groups. Finally, only national statistics are provided to highlight the diseases of AI/ANs. Certain states do not have high proportions of AI/ANs populations and might not have identified this group for targeting when addressing infectious diseases. Jurisdictions with concentrated populations of AI/ANs might analyze their surveillance data routinely and address concerns specific to this population.

Surveillance of notifiable diseases is essential for the prevention and control of infectious diseases. Health status assessments for AI/ANs often are hindered by a lack of complete and accurate data regarding race in surveillance systems (5). AI/ANs have lower socioeconomic status overall, and although those who live on reservations and tribal members have access to Indian Health Service hospital services, not all AI/ANs might have ready access to health care. State and local health departments with large segments of AI/AN populations have opportunities to develop efficient intervention efforts and programs tailored to this population (8).

## Figures and Tables

**TABLE:** Number and incidence rate per 100,000 population for 26 selected notifiable diseases, by American Indian/Alaska Native (AI/AN), black, or white race — United States, 2007–2011

Disease	AI/ANs		Blacks		Whites		Total		Rate ratio: AI/ANs compared with whites	% with no race identified
			
	No.	Rate	No.	Rate	No.	Rate	No.	Rate		
Botulism, foodborne	26	0.12	28	0.03	339	0.03	672	0.04	4.38	35.42
Chickenpox (varicella)	503	2.45	7,086	8.54	7,8776	6.45	110,634	7.22	0.38	17.82
*Chlamydia trachomatis*	77,092	374.93	2,189,748	2,639.03	1,841,172	150.74	6,283,761	409.90	2.49	0.30
Cryptosporidiosis	223	1.08	3,202	3.86	29,010	2.38	45,721	2.98	0.46	25.63
Ehrlichiosis, total	219	1.07	167	0.20	7,250	0.59	12,348	0.81	1.79	36.46
Gonorrhea	12,764	62.08	894,198	1,077.66	317,271	25.97	4,825,097	314.75	2.39	7.31
Giardiasis	385	1.87	6,875	8.29	38,506	3.15	93,164	6.08	0.59	41.55
*Haemophilus influenzae*	206	1.00	1,822	2.20	9,340	0.76	14,990	0.98	1.31	20.69
Hantavirus pulmonary syndrome	20	0.10	1	0.00	77	0.01	112	0.01	15.43	10.71
Hepatitis A, viral acute	66	0.32	677	0.82	5,607	0.46	10,544	0.69	0.70	28.15
Hepatitis B, viral acute	144	0.70	3,532	4.26	9,433	0.77	184,114	12.01	0.91	2.22
Hepatitis C, viral acute	88	0.43	261	0.31	3,220	0.26	4,553	0.30	1.62	19.33
Legionellosis	42	0.20	2,890	3.48	10,590	0.87	16,870	1.10	0.24	16.87
Lyme disease	476	2.31	1,649	1.99	85,721	7.02	160,209	10.45	0.33	38.68
Meningococcal disease	48	0.23	707	0.85	2,899	0.24	4,776	0.31	0.98	18.91
Pertussis	788	3.83	3,709	4.47	57,644	4.72	85,723	5.59	0.81	23.48
Salmonellosis	1,783	8.67	21,647	26.09	142,495	11.67	252,169	16.45	0.74	28.99
Shiga toxin–producing *Escherichia coli*	161	0.78	1,020	1.23	16,749	1.37	26,058	1.70	0.57	27.09
Shigellosis	1,115	5.42	17,822	21.48	37,309	3.05	85,172	5.56	1.78	28.71
Spotted fever rickettsiosis	519	2.52	434	0.52	7,325	0.60	11,108	0.72	4.21	23.17
*Streptococcus pneumoniae*, invasive (all ages)	575	2.80	8,652	10.67	28,766	2.36	49,548	3.23	1.19	20.76
*Streptococcus pneumoniae*, invasive (age <5 years)	297	20.02	2,249	48.85	9,214	15.03	16,102	19.94	1.33	21.96
Syphilis, primary and secondary	367	1.78	31,469	37.93	28,046	2.30	66,707	4.35	0.78	4.00
Tuberculosis	813	3.95	15,167	18.28	25,944	2.12	59,458	3.88	1.86	0.77
Tularemia	47	0.23	15	0.02	413	0.03	626	0.04	6.76	21.73
West Nile virus disease	184	0.89	348	0.42	5,142	0.42	6,418	0.42	2.13	25.33
**Total**	**98,767**	**—**	**3,215,027**	**—**	**2,793,116**	**—**	**12,420,236**	**—**	**—**	**—**
